# Liquid water might not be the only answer: Evaluating pore-filling materials in the Martian crust

**DOI:** 10.1073/pnas.2503071122

**Published:** 2025-08-18

**Authors:** Wanbo Xiao, Lu Pan, Yanbin Wang, Jiaqi Li

**Affiliations:** ^a^SKLab-DeepMinE, MOEKLab of Orogenic Belts and Crustal Evolution, School of Earth and Space Sciences, Peking University, Beijing 100871, China; ^b^School of Earth and Space Sciences, University of Science and Technology of China, Hefei, Anhui 230026, China; ^c^Deep Space Exploration Laboratory, Hefei, Anhui 230026, China

Liquid water was once abundant on the Martian surface. However, most of Mars’ water has been lost due to atmospheric escape (1) or deep burial (2), leaving a small fraction as surface ice today. The presence of liquid water in the subsurface remains uncertain. Analysis of marsquake coda waves suggests that the lowland crust, traversed by seismic waves, is predominantly dry (3). In contrast, Wright et al. (4) integrated seismological and gravity data (5, 6) with petrophysical modeling and proposed that the Martian mid-crust (11 to 20 km) beneath the InSight (7) landing site, in the northern lowlands, can be best explained by igneous rocks with liquid water filling fractures.

**Fig. 1.**
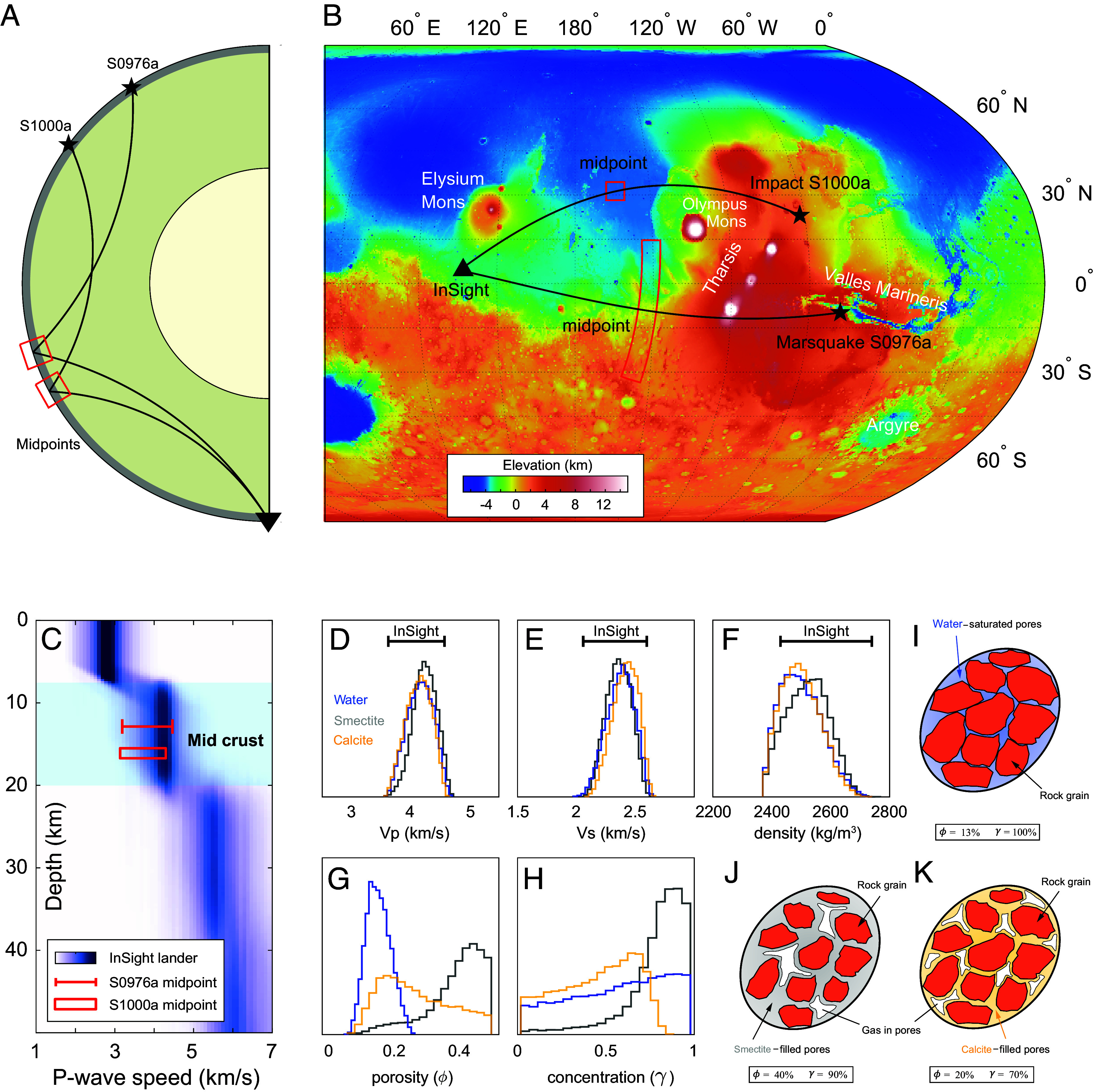
(*A*) Seismic ray paths for the reflected waves that probe the structures at the midpoints. (*B*) Topographic map of Mars showing the InSight lander, marsquake S0976a, and impact S1000a epicenters along with their midpoints (3,500 to 4,500 km away from InSight). (*C*) P-wave speed profile at the InSight landing site, adapted from Knapmeyer-Endrun et al. (5) and Wright et al. (4), with additional constraints on midpoint velocities from this study (shown as red bar and box). (*D*) Comparison between observed P-wave speed at InSight (horizontal error bar) and predicted probability density functions (PDFs) for three potential pore-filling materials: liquid water, smectite, and calcite. (*E*–*H*) Similar probabilistic comparisons for the predicted S-wave speed, bulk density, porosity, and pore concentration, respectively. (*I*) Lithological model of the Martian mid-crust, with water-saturated pores [13% porosity; Wright et al. (4)]. (*J*) Alternative model with smectite-cemented pores (40% porosity, 90% smectite, and 10% gas). (*K*) Alternative model with calcite-cemented pores (20% porosity, 70% calcite, and 30% gas).

To evaluate the interpretation by Wright et al. (3), three key factors should be considered. First, liquid water is not the only possible pore-filling material—solid cementations, such as secondary minerals, may also explain the geophysical observations. Second, Palin et al. (8) proposed that hydrated igneous rocks would have undergone metamorphic transitions to prehnite–pumpellyite (PP) or pumpellyite–actinolite (PA) facies, altering their physical properties. Third, fully understanding Mars’ water cycle requires extending the investigation beyond the InSight landing site (Fig. 1 *A* and *B*).

We expanded the analysis to include alternative pore-filling materials (9), using smectite and calcite as endmembers representing hydrated and anhydrous secondary minerals with relatively low and high elastic moduli, respectively. Our results demonstrate that these materials can equally explain the observations (Fig. 1 *D*–*K*), challenging the assumption that liquid water is the dominant pore-filling phase.

We further examined the effects of basalt transforming into PP or PA facies. If the Martian mid-crust is fully transformed into metamorphic phases, the presence of liquid water becomes less likely. This is because the mineralogical changes reduce the rock’s density and seismic wave speed, lessening the need for additional pore-filling materials, including water, to achieve such reductions. However, due to kinetic limitations, the transformation may be incomplete, leaving primary basaltic phases intact. In this case, pore spaces are required to account for geophysical observations, but distinguishing between liquid water and solid pore-filling materials (e.g., secondary minerals) remains challenging.

Beyond the InSight landing site, we applied a technique (10) to probe the crust at the midpoint between the seismic source (marsquakes or meteorite impacts) and the station, approximately 3,500 to 4,500 km away (Fig. 1*A*). The seismic wave speeds at these midpoints are 7 to 10% lower than those at the InSight landing site in the Martian mid-crust (Fig. 1*C*). Although such a decrease in seismic wave speeds may appear more indicative of liquid water, petrophysical modeling results suggest that solid pore-filling materials could also account for these observations, indicating that liquid water is not the only viable pore-filling candidate at these locations.

Seismic wave speeds and density data alone cannot uniquely identify liquid water in the Martian mid-crust, given the consideration of alternative pore-filling materials. This nonuniqueness extends beyond the InSight landing site to other regions in the northern lowlands and near the dichotomy boundary. Future missions targeting the southern highlands, where both crustal structures and deep hydrological conditions remain unknown (5), could provide key insights into the global distribution of subsurface water and the evolution of Mars’ hydrosphere.
